# Supplementary material for: Late adolescents’ own and assumed parental preferences towards health-care related confidentiality and consent in Belgium
Source: PLoS One. 2021 Jun 2;16(6):e0252618. doi: 10.1371/journal.pone.0252618 (PMC8171959; doi:10.1371/journal.pone.0252618)
Supplement: S1 Appendix — (DOCX) [file pone.0252618.s001.docx]

**S1 Appendix.** English translation of vignettes

***Vignette 1***

*Imagine that you are an adolescent aged 15 years old. You must go to the intensive care unit for a medical problem.*

You are out with a couple of friends. You have had a little too much to drink that night and you fall on the ground. By a stupid coincidence, you fall on a shard of glass, and you now have a cut in your hand. You are brought to the intensive care unit where a physician treats your injury. You realize that you will get in trouble when your parents find out you were ‘drunk’. You ask the attending physician not to inform your parents about your ‘drunkenness’. You will tell your parents that you tripped and hurt your hand this way.

Is it your opinion that the physician should report the ‘drunkenness’ to your parents, despite your express request not to do so?

**O** Yes

**O** No

What would be the opinion of your biological or adoptive parents? Would they feel that the physician should report your ‘drunkenness’ to them, despite your express request not to do so?

**O** Yes

**O** No

***Vignette 2***

*Imagine that you are an adolescent aged 15 years old. You have an appointment with an oral specialist. This is related to the position of your teeth.*

You have a so-called ‘underbite’. Your lower jaw is too far backwards. This is a strange sight, and you receive a lot of teasing at school because of it. The physician says that surgery is the only way to straighten your teeth, and that braces will not help. For this, the position of your lower jaw must be corrected. Because you value your looks, you would like to undergo this surgery. You hope that the teasing will then stop. However, your parents believe that you look fine, and that surgery is dangerous and redundant.

Is it your opinion that you should be allowed to make your own independent decision on this surgery?

O Yes

O No

What would be the opinion of your biological or adoptive parents? Would they feel that you should be allowed to make your own decision on this surgery?

O Yes

O No

***Vignette 3***

*Imagine that you are an adolescent aged 15 years old. You have a medical problem for which you consult your family physician.*

You have recently gotten into a new romantic relationship, but you have an annoying medical issue. Your physician has diagnosed you with a sexually transmitted disease (STD), one which is easily treatable. When you and your partner are treated with a course of antibiotics, the problem will be solved. You realize that you will get in trouble when your parents hear about this infection. You ask the physician not to tell your parents.

Is it your opinion that the physician should inform your parents about your STD, despite your express request not to do so?

O Yes

O No

What would be the opinion of your biological or adoptive parents? Would they feel that the physician should report your STD to them, despite your express request not to do so?

O Yes

O No

***Vignette 4***

*Imagine that you are a (female) adolescent aged 15 years old. You have a medical problem for which you consult your family physician.*

You have been getting your period since about a year. Every month you experience tremendous menstrual pains, and you would like to do something about it since the intense stomach aches prevent you from playing sports or exercising your hobby. The family physician suggests a hormonal contraceptive pill as treatment for these painful menstrual pains. You think that it a good solution and you would like to try this. Your parents feel that a 15-year old girl should not be taking ‘the pill’ and do not agree to this treatment. Unfortunately, there are no other effective treatments available.

Is it your opinion that the physician should prescribe the pill, despite the parents’ objections?

O Yes

O No

What would be the opinion of your biological or adoptive parents? Would they feel that the physician should prescribe the pill despite the fact that they do not agree with the treatment?

O Yes

O No
