# Supplementary material for: Late adolescents’ own and assumed parental preferences towards health-care related confidentiality and consent in Belgium
Source: PLoS One. 2021 Jun 2;16(6):e0252618. doi: 10.1371/journal.pone.0252618 (PMC8171959; doi:10.1371/journal.pone.0252618)
Supplement: S2 Appendix — (DOCX) [file pone.0252618.s002.docx]

**S2 Appendix.** Dutch version of the vignettes used in this study

1. Beoordeel de volgende vier casussen.

**Casus 1**

*Beeld je in dat je een tiener bent van 15 jaar oud. Je moet naar de dienst spoedgevallen voor een medisch probleem.*

Je gaat uit met een paar vrienden. Je hebt die avond iets teveel gedronken en je bent op de grond gevallen. Door stom toeval ben je in een glasscherf terecht gekomen, daardoor heb je een snede in je hand. Je wordt naar de spoedafdeling gebracht waar de arts de wonde verzorgt. Je beseft dat je in de problemen zal komen wanneer je ouders horen dat je ‘dronken’ was. Je vraagt de behandelende arts je ouders niet in te lichten over je ‘dronkenschap’. Je zal aan je ouders vertellen dat je gestruikeld bent en zo je hand hebt gekwetst.

Vind je dat de arts het recht heeft om je ‘dronkenschap’ aan je ouders te melden, ondanks jouw uitdrukkelijk verzoek om dat niet te doen?

**O** Ja

**O** Nee

Wat zou het standpunt zijn van je biologische of adoptie-ouders? Zouden zij vinden dat de arts je ‘dronkenschap’ aan hen moet melden, ondanks jouw uitdrukkelijk verzoek om dat niet te doen?

**O** Ja

**O** Nee

**Casus 2**

*Beeld je in dat je een tiener bent van 15 jaar oud. Je hebt een afspraak bij een mond- en kaak- specialist. Dat heeft te maken met de stand van jouw tanden.*

Je hebt een bijzondere positie van je onderkaak, een zogenaamde ‘onderbeet’. Je onderkaak staat teveel naar achter. Dat is een vreemd zicht, en daardoor word je vaak gepest door jouw leeftijdsgenoten. Volgens de dokter is een operatie de enige manier om jouw tanden recht te zetten, een beugel zal hier niet helpen. Daarvoor moet je onderkaak doorgezaagd worden. Omdat je veel belang hecht aan je uiterlijk, wil je die behandeling graag laten uitvoeren. Je hoopt dat het pesten dan zal stoppen. Je ouders vinden echter dat je er goed uitziet, en dat een operatie gevaarlijk en overbodig is.

Vind je dat je zelf en zelfstandig mag beslissen over deze operatie?

**O** Ja

**O** Nee

Wat zou het standpunt zijn van je biologische of adoptie-ouders? Zouden zij vinden dat je zelf en zelfstandig mag beslissen over deze operatie?

**O** Ja

**O** Nee

**Casus 3**

*Beeld je in dat je een tiener bent van 15 jaar oud. Je hebt een medisch probleem en daarvoor ga je naar de huisarts.*

Je hebt sinds kort een nieuwe relatie en hebt een vervelend probleem. De dokter stelde een seksueel overdraagbare aandoening (SOA) vast, die gelukkig wel snel en goed te behandelen is. Wanneer jij en je vriend(in) worden behandeld met een korte kuur antibiotica, zal het probleem opgelost zijn. Je beseft dat je in de problemen zal komen wanneer je ouders horen dat je ‘dronken’ was. Je beseft dat je in de problemen zal komen wanneer je ouders iets over deze infectie te horen krijgen. Je vraagt aan de dokter om niets aan je ouders te zeggen.

Vind je dat de arts het recht heeft om je SOA aan je ouders te melden, ondanks jouw uitdrukkelijk verzoek om dat niet te doen?

**O** Ja

**O** Nee

Wat zou het standpunt zijn van je biologische of adoptie-ouders? Zouden zij vinden dat de arts het recht heeft om je SOA aan hen te melden, ondanks jouw uitdrukkelijk verzoek om dat niet te doen?

**O** Ja

**O** Nee

**Casus 4 (opmerking: bij deze casus moeten de mannen zich even inbeelden dat ze een vrouw zijn …)**

*Beeld je in dat je een tiener bent van 15 jaar oud. Je hebt een medisch probleem en daarvoor ga je naar de huisarts.*

Je hebt sinds ongeveer één jaar je maandstonden. Je hebt daar elke maand veel last van, en je zou daar graag iets laten aan doen. De felle buikpijn belemmert je sport- en hobbybeoefening. De huisarts stelt een hormonale anticonceptiepil voor als behandeling om de pijnlijke maandstonden te doen afnemen. Je vindt dit een goede oplossing en wil dat proberen. Jouw ouders vinden echter dat een meisje van 15 jaar ‘de pil’ niet hoort te nemen en gaan niet akkoord met deze behandeling. Er zijn helaas geen andere effectieve behandelingen beschikbaar.

Vind je dat de dokter de pil mag voorschrijven, ondanks de mening van de ouders?

**O** Ja

**O** Nee

Wat zou het standpunt zijn van je biologische of adoptie-ouders? Zouden zij vinden dat de dokter de pil mag voorschrijven ondanks het feit dat zij niet akkoord gaan met deze behandeling?

**O** Ja

**O** Nee
