# Supplementary material for: Late adolescents’ own and assumed parental preferences towards health-care related confidentiality and consent in Belgium
Source: PLoS One. 2021 Jun 2;16(6):e0252618. doi: 10.1371/journal.pone.0252618 (PMC8171959; doi:10.1371/journal.pone.0252618)
Supplement: S1 Table — (DOCX) [file pone.0252618.s003.docx]

**S1 Table. Descriptive overview of vignette answers (in %).**

|  |  | No | Yes |
| --- | --- | --- | --- |
| Confidentiality | Drunk_a_ | 37.1 | 62.9 |
|  | Drunk_p_ | 11.8 | 88.2 |
|  | Std_a_ | 21.5 | 78.5 |
|  | Std_p_ | 36.6 | 63.4 |
| Consent | Surgery_a_ | 67.7 | 32.3 |
|  | Surgery_p_ | 28.1 | 71.9 |
|  | Pill_a_ | 22.5 | 77.5 |
|  | Pill_p_ | 54.8 | 45.2 |

Note. The subscript a refers to respondents’ own preferences. The subscript p refers to assumed parental preferences.
