# Supplementary material for: Late adolescents’ own and assumed parental preferences towards health-care related confidentiality and consent in Belgium
Source: PLoS One. 2021 Jun 2;16(6):e0252618. doi: 10.1371/journal.pone.0252618 (PMC8171959; doi:10.1371/journal.pone.0252618)
Supplement: Attachment — Submitted filename: Response to Reviewers.docx [file pone.0252618.s005.docx]

Dear Editor,

Dear Prof. Menezes,

Thank you for your feedback on our paper, which was very helpful. We would also like to thank both reviewers for their positive feedback on the newest version of our manuscript. In this letter, we provide a point-by-point response to each of the remaining comments and suggestions you and the reviewers provided. We hope that this version can be reconsidered for publication in *PLOS One*.

With warm regards,

David

**Editor/journal comments**

**Comment 1:** Kindly note that some of the comments made by the reviewers on the previous version are not appropriately addressed in the revised version. Please do have a relook at the previous set of comments in addition to the new set of comments.

**Response:** Thank you for this comment. We have revisited the earlier comments from the reviewers and have made some additional revisions with regards to the tense of our manuscript. Aside from this, we believe we have thoroughly addressed all comments from the reviewers – but are willing to revisit specific points if need be.

**Reviewer 1**

**Comment 1:** I want to congratulate the authors on the significant improvement of the paper. The authors addressed all comments from the review in scientifically sound and intelligible way. The paper is now suitable for publication. There are only few very small comments that need to be addressed before publishing.
**Response:** Thank you for your positive feedback on the revised version of our manuscript. Below, you can find a point-by-point response to your minor comments. We hope this version can be accepted for publication in Plos One.

**Minor comments:**

**Comment 2:** Lines 191-192, 195-200: The description of analyses should be placed in the Methods section, under the Data analysis header. The results of analyses should remain in the Results section.

**Response:** In line with your comments, we have now created a new header ‘Data analysis’ (just before the Results section, still in the Methods section), which contains the information from the lines you highlighted.
**Comment 3:** Line 271: a word is missing (contraceptive use, the is more pronounced as respondents prefer physicians to respect their preferences).

**Response:** Thank you for noticing this! You are quite right – the word ‘difference’ was missing. We have added it.

**Comment 4:** You don’t need to provide the full questionnaire in Dutch, it is sufficient to have the section that was described in the paper, in English.

**Response:** We have removed the superfluous sections of the survey that were provided, and now only present the Dutch version of the vignettes in the supplemental materials.

**Reviewer 2**

**Comment 1:** Thank you for the opportunity to review this revised paper regarding adolescents´ own and assumed parental preferences regarding confidentiality and consent in Belgium. This is an important and well-performed study dealing with a clinically important and difficult issue when treating adolescents. The authors manuscript has improved significantly, and I have only minor comments.

**Response:** Thank you for your positive feedback on the revised version of our manuscript. Below, you can find a point-by-point response to your minor comments. We hope this version can be accepted for publication in Plos One.

**Comment 2:** In the abstract, methods and results are still written in present tense and not past tense as in the main manuscript.

**Response:** We have revised the tense in the ‘Methods’ and ‘Results’ sections in the abstract.

**Background**

**Comment 3:** Line 80-85: you state that evidence from Europe is lacking. However, Thomsen et al have looked at parents’ ambivalent feelings regarding confidentiality in a paper from 2019. The reference from Thomsen, that you do cite is dealing with guidelines for adolescent friendly health services and not only confidentiality and consent.
**Response:** Thank you for drawing further attention to this article. We have nuanced our statement regarding the lack of European studies, we now state that European studies are scarce, and highlight Thomsen et al.’s study as one of the exceptions.

**Comment 4:** Line 96-98: I recommend you to use more recent guidelines regarding time alone during consultations.
**Response:** We have now also cited two other articles that were already included in our reference list which are more recent (2006 and 2018) that echo the statement in the sentence.

**Comment 5:** Line 105-109:Again I recommend newer references e.g.Mcdonagh et al, Acta Paediatrica 2006
**Response:** Thank you for this suggestion, we have included the reference to McDonagh et al. (2006) and have also included another recent study from Wright et al. (2017) published in Future Healthcare Journal.

**Comment 6:** Line 113+: the aim of the study should probably be in past tense

**Response:** We have revised this paragraph and put it in past tense where appropriate.

**Comment 7:** Line 123-131: I think that this highly relevant paragraph should be moved up in the background section

**Response:** Thank you for your thoughtful comments about this paragraph. We certainly agree with you that this paragraph could work higher in the background section, but we struggled to find a good place for it as it balances between methodology and background. In a way, we are arguing how our study participants (a group that we have not yet introduced in the beginning of the background) can be characterised as ‘late adolescents’. We fear that moving it up too high may be confusing for readers. We have moved it above the aim of the study and believe these last two paragraphs now read a little easier.

**Materials and methods**

**Comment 8:** Line 139: I guess the students were asked to participate in the study and not in class?

**Response:** Indeed – we have clarified this sentence.

**Comment 9:** Line 142-147: I think this should be moved to an Ethics section
I miss a statistics section and suggest that you re-phrase and move the paragraph line 191-200 from the results section

**Response:** Thank you for these comments. Regarding your comments about the ethics section, Plos One submission guidelines state:

Methods sections of papers on research using human subjects or samples must include ethics statements that specify:

- **The name of the approving institutional review board or equivalent committee(s)**. If approval was not obtained, the authors must provide a detailed statement explaining why it was not needed
- **Whether informed consent was written or oral**. If informed consent was oral, it must be stated in the manuscript:
  - Why written consent could not be obtained
  - That the Institutional Review Board (IRB) approved use of oral consent
  - How oral consent was documented

Thus, it appears that the statement about ethics must be included in the Methods section, as currently the case.

Regarding the point about line 191-200 (mirroring comments from Reviewer 1), we have created a new header ‘Data analysis’ (just before the Results section, still in the Methods section), which contains the information from the lines you highlighted.

**Results**

**Comment 10:** Line 205: please provide a more descriptive headline than T test results.

**Response:** We have changed this subtitle to ‘Differences in confidentiality and consent preferences’ and changed the ‘binomial logistic regression’ subtitle to ‘A closer look at late adolescents’ preferences’.

**Comment 11:** Line 209-10: You mention SD in the ‘drunk’ case

**Response:** Following your next comment, we have now removed all numbers in this paragraph.

**Comment 12:** Line 206-218: This section is a repetition from the table 3 and I think you could omit the numbers and refer to the table. Furthermore, I think that your explanation in your reply to the reviewers regarding how you get the results should be included in the text or table legend as it makes it much more easy to understand.

**Response:** Following your suggestion, we have now removed the numbers from the text and refer to Table 3 – which has increased the readability of the paragraph. We have also included part of the reply from the comments to the reviewers to the note of Table 3, hoping to clarify some of the confusion regarding the coding of the vignettes.

**Comment 13:** Line 223: I think it is an overstatement to say that there is limited significant differences as it only applies to the ‘surgery case’ and this could be ‘by chance’. If you think the difference is true, I suggest you discuss this in the discussion section as it is quite interesting.
**Response:** We agree with your assessment regarding this finding. We have nuanced this finding further, and already discuss these potential gender differences in the discussion (line 286-293). However, we believe more research is needed to draw clear conclusions about such gender differences – our study cannot do so.

**Comment 14:**

Line232: we present (present tense)
Line 236-237: believed (past tense)
Line 240 supported (past tense)

**Response:** We have revised all these errors – thank you for noticing them.

**Discussion**

**Comment 15:** Line 253: it seems that there are words missing e.g. ‘perceptions between….’

**Response:** We agree that this sentence ended rather abruptly, and we have extended it somewhat so that it flows better within the text.

**Comment 16:** Line 262: I would use the reference by Thomsen et al describing parents’ ambivalent views

**Response:** The study by Thomsen et al. is number 35 in our reference list, so we were already including it in the references for this sentence. Thank you for suggesting it!

**Comment 17:** Line 271: is there a word missing?

**Response:** Thank you for noticing this! You are quite right – the word ‘difference’ was missing. We have added it.

**Conclusion**

**Comment 18:** Line 322: regardless of parental consent?

**Response:** We have added ‘parental’ to this sentence.
