# Supplementary material for: Late adolescents’ own and assumed parental preferences towards health-care related confidentiality and consent in Belgium
Source: PLoS One. 2021 Jun 2;16(6):e0252618. doi: 10.1371/journal.pone.0252618 (PMC8171959; doi:10.1371/journal.pone.0252618)
Supplement: Attachment — Submitted filename: Response to Reviewers.docx [file pone.0252618.s006.docx]

Dear Editor,

Dear Prof. Menezes,

Thank you for your feedback on our paper, which was very helpful. We would also like to thank both reviewers for their positive feedback on the newest version of our manuscript. In this letter, we provide a point-by-point response to each of the remaining comments and suggestions you and Reviewer 2 provided. We hope that this version can be reconsidered for publication in *PLOS One*.

With warm regards,

David

**Editor comments**

**Comment 1:** Abstract: Delete the last sentence from the conclusion sub-section.

**Response:** Sentence has been removed.

**Comment 2:** Introduction-“Generally, sexual and reproductive health conditions often do not require consent from a parent or guardian.”: Cite a reference.

**Response:** We now cite reference at the end of this sentence.

**Comment 3:** Introduction-Lines 52 & 73 (Please note that the line numbers refer to the marked revised manuscript file): Why is ‘’late’’ mentioned within small brackets?

**Response:** We removed the small brackets around “late” in line 73, we removed (late) entirely in line 52.

**Comment 4:** Introduction-Lines 82-83: Syntax of the sentence needs to be addressed.

**Response:** We have revised this sentence.

**Comment 5:** Introduction-Line 83-“Thomson et al. (2019)’’: Cite the reference as per journal style.

**Response:** We have revised this sentence entirely.

**Comment 6:** Introduction-Line 99: Cite the reference as per journal style.

**Response:** Amended.

**Comment 7:** Introduction-Line 125-‘’KU’’: Provide the full form as well.

**Response:** There is no full form, the official name of the university is KU Leuven. The KU used to stand for something, but today the institution only uses the abbreviation and never uses the full form.

**Comment 8:** Introduction-Lines 113-125: Further add on to mention that ‘’late adolescence” refers to teenage years between the ages of 15 and 19. Cite an appropriate reference to support this statement [possibly, a UNICEF related reference (<https://www.unicef.org/sowc2011/pdfs/Early-and-late-adolescence.pdf)>].

**Response:** We have added this UNICEF-reference.

**Comment 9:** Methods-Line 157: It is irrelevant to mention ‘’experiments where humans were involved’’ here. Isn’t it?

**Response:** But we do mention this, no? Or do you mean to say we should remove this?

**Comment 10:** Methods-Lines 160-161: Replace ‘’(sometimes also referred to as ‘cases’ in the rest of this article)’’ with ‘’(cases)’’.

**Response:** Done.

**Comment 11:** Methods-Lines 165-167: In addition, specify the related status in Belgium.

**Response:** We have added this info.

**Comment 12:** Methods-Line 181: Replace “case’’ with ‘’cases’’.

Methods-Line 182: Replace ‘’case’’ with ‘’cases’’.

**Response:** Done.

**Comment 13:** Methods-Lines 188-192: Would you like to mention the reason for not obtaining information on father’s educational status?

**Response:** Previous research has shown that mother’s educational attainment is a robust indicator for socioeconomic status of adolescents. However, given the limited impact of sociodemographic indicators in this analysis, we decided not to spend too many words on this point.

**Comment 14:** Results-Line 205: I would prefer to consider using the term ‘’’woman” for an adult female and not an adolescent female. It is not mandatory to revise this accordingly. However, since you have used the term ‘’boys’’ and ‘’girls” in Table 5, it would be better to replace ‘’women’’ with ‘’girls’’.

**Response:** This has been amended.

**Comment 15:** Results-Lines 207-208: It is mentioned that over 95% of the sample was between 17 and 19 years of age. Retain this sentence. In addition, mention the age range (17 to 23) in the text and not at the bottom of Table 1. Provide the mean (standard deviation) for age in the text and not in Table 1. In Table 1, provide the number (%) for age 17, age 18, age 19, ages 20-23.

**Response:** We’ve made the necessary changes to table 1 and in the text directly preceding this table.

**Comment 16:** Results: If I am not mistaken, Table 2 is not referred to in the text.

**Response:** We did refer to Table 2 in line 209 (now 201).

**Comment 17:** Table 2: In the footnote denote the case numbers for drunk/std/pill/surgery.

**Response:** Done.

**Comment 18:** Table 3: Indicate the case number in brackets. For example: Drunk (Case 1)

**Response:** Done.

**Comment 19:** Table 4: In the footnote denote the case numbers for drunk/std/pill/surgery.

**Response:** Done.

**Comment 20:** Table 5: Indicate the case number in brackets. For example: Drunk (Case 1).

**Response:** Done.

**Comment 21:** Discussion/Conclusion: Wherever applicable, replace ‘’adolescents’’ with ‘’late adolescents’’. For your information: Early adolescence (10-14 years); Late adolescence (15-19 years). I wish participants 20 and above (age range of participants in the present study: 17-23) were not included in the present study (or their responses were not considered for analysis) in the first place.

**Response:** We have gone through both the discussion and conclusion and replaced “adolescents” with “late adolescents” in several instances – a few were left as they were, since they did not refer to adolescents in our study but rather referred to other studies. We appreciate your comments in this regard and will keep this in mind in future studies on the topic.

**Comment 22:** Besides, address the comments made by the reviewer(s).

**Response:** All comments by the reviewer have been addressed. Thank you for your useful points.

**Reviewer 1**

**No Comments.**

**Reviewer 2**

**Comment 1:** Congratulations with the revision of the manuscript. I find it ready for submission after only a few minor comments and suggestions.

**Response:** We thank you for your positive feedback, and hope that our point-by-point overview of the changes made in accordance with your suggestions is sufficient to accept this paper for publication in Plos One. We thank you for your useful suggestions throughout the review process.

**Comment 2:** The references in the manuscript are not in order starting with 1, 2, 36, 18 etc.

**Response:** The order has now been updated.

**Comment 3:** Reference 35 is still the wrong one from Thomsen et al. The correct reference must be: Thomsen EL et al. Int J Adolesc Med Health 2019. doi: 10.1515/ijamh-2018-0226. Online ahead of print.

**Response:** We’ve swapped this out with the correct reference now.

**Comment 4:** Line 52: I would erase (late) as I think that health professionals struggle with providing care to adolescents (10-19) and not only late adolescents.

**Response:** This has been amended.

**Comment 5:** Line 171: I think this sentence is ambiguous as I believe you assess whether adolescents prefer that physicians inform the parents and not whether they think this is within the legal framework.

**Response:** Your assessment is correct, but we do emphasize in this sentence that respondents had to indicate if they were ‘of the opinion’ the physician should inform the parents, which should signal that it was their opinion that was measured instead of their assessment of the legal regulations.

**Comment 6:** Line 187: I would erase ‘in the top left and bottom right quadrants as it is difficult to understand if you do not refer to the table. Furthermore, I would erase ‘In what follows’ as this is unnecessary.

**Response:** Both changes have been carried out.

**Comment 7:** Table 5: the lines are difficult to follow.

**Response:** We appreciate that this may seem confusing, but we hope that with the copy-editing team of Plos One can make this look much clearer in the published article. If further changes need to be made, we’d be happy to do so.

**Comment 8:** Line 257: I would prefer ‘preference’ instead of perceptions.

**Response:** This has been amended.
